# Special Issue: Advances in Plant Virus Diseases and Virus-Induced Resistance

**DOI:** 10.3390/ijms26167748

**Published:** 2025-08-11

**Authors:** Pedro Martínez-Gómez

**Affiliations:** Department of Plant Breeding, Centro de Edafología y Biología Aplicada del Segura-Consejo Superior de Investigaciones Científicas (CEBAS-CSIC), P.O. Box 164, 30100 Murcia, Spain; pmartinez@cebas.csic.es; Tel.: +34-968-396-200

After fungi, viruses are the most important plant pathogens. Plant viruses cause significant losses in agricultural crops worldwide, affecting the yield and quality of agricultural products [1], with an estimated burden of approximately USD 30 billion per year [2]. Plants are affected by numerous viruses and viroids that are linked to vegetative propagation practices in many cultivated species, with rapid virus transmission by natural vectors being the main form of plant pathogen [3]. Viruses are also responsible for most emerging plant diseases that are associated with crop intensification, global trade, and the limited number of tools for controlling viral vectors [4]. Factors associated with the increasing emergence of viral diseases include crop intensification, global trade, limited tools for controlling viral vectors, and, undoubtedly, climate change. The different forms of mutation, recombination, and other types of genetic exchange, which are considered as the basis of the evolutionary forces of viruses, have undoubtedly given rise to the genetic diversity found in plant virus populations. In this context, environmental factors play an important role in driving virus evolution. In addition, the rapid expansion of human activity in the world of commerce, agriculture, the anthropization of natural ecosystems, and climate change has further contributed to the instability between hosts and virus populations, favoring the emergence of viruses with mutant and/or recombinant forms, with potentially negative impacts on plants, vectors, and ecosystems [5]. New studies will explore the ecology of vectors and viruses in natural conditions to understand how they emerge, spread, and adapt [6]. Understanding their biology, infection dynamics under changing environments, or interactions with other hosts and pathogens will be crucial for developing effective and sustainable strategies to mitigate the impact of plant viruses in agriculture [2]. In this pandemic context, different works deal with recent advances and perspectives in the study of viruses and viroids that affect plant species, mainly with regard to the detection and characterization of the viruses and viroids involved, their transmission, an analysis of their pathogenicity, and the search for novel genetic control tools [7]. Modern prevention, diagnosis, and recovery of planting material methods are at the forefront of the fight against viral infection.

Regarding virus diagnosis, new sequencing technologies are rapidly reshaping the way in which we can identify and characterize new viruses and viroid isolates. This information is usually deposited in databases. Researchers can comprehensively understand virus–host interactions by integrating data mining with other omics data, such as proteomics (the study of proteins) and metabolomics (the study of metabolic processes). Recent progress has significantly enhanced the efficiency and accuracy of virus identification by using a sophisticated next-generation sequencing (NGS) data mining approach. This study provides an in-depth discussion of these techniques, offering a detailed overview of workflows and applicable computational methods [8]. These high-throughput technologies have also been used to obtain more in-depth knowledge relating to the pathogenicity mechanisms that underlie the interactions between plants and their main viruses and viroids. Feng et al. [9] showed that tomato (*Solanum lycopersicum* L.) virus—Tomato spotted wilt orthotospovirus (TSWV)—may engage in long-distance movements via the xylem. According to the high-throughput proteomic analysis, a total of 3305 proteins were identified in two groups. Among them, 315 host proteins (163 upregulated and 152 downregulated) and three viral structural proteins (N, Gn, and Gc) were found to be differentially expressed in the two groups. This comparative proteomic analyses of *Nicotiana tabacum* xylem sap under TSWV stress helps us to understand the host plant xylem system response to TSWV infection, providing an important basis for the further study of the mechanism of TSWV long-distance movements in host plant vascular systems.

On the one hand, in the study of virus-induced resistance, new approaches include molecular plant–virus interactions to identify new breeding targets. Recent studies have described the epigenetic regulation of this response and the possible role in virus-triggered induced resistance [10]. These authors identified genes that were directly targeted by the RdDM-related RNA Polymerase V (POLV) complex and the histone demethylase protein JUMONJI14 (JMJ14) during infection. Independently of the studied genes, the authors suggested that plants previously stimulated with viruses, such as the turnip mosaic virus (TuMV), displayed a higher resistance to later virus infections. Another example was also addressed by other authors, suggesting that the ‘Garrigues’ almond (*Prunus dulcis* (Miller) D.A. Webb) variety triggers a complex defense response in peach (*Prunus persica* (L.) Batsch) rootstock, potentially involving the interplay of epigenetic modifications and small RNA-mediated priming of antiviral defenses, which may ultimately contribute to Plum pox virus (PPV) resistance [11]. The possibilities of increasing plant resistance by stimulating their immune system have expanded, which results from in-depth studies exploring the molecular and genetic bases of plant resistance toward viruses, as well as investigations into the mechanisms of induction of protective reactions in the plant organism. Maksimov et al. [12] describe modern approaches to plant protection against viruses via genome editing, the regulation of the expression of the host plant and/or viral genes via RNA interference, and the formation of an artificial consortium of plants with rhizospheric and/or endophytic microorganisms that combine protective activity and immunomodulating potential. In addition, recently developed biotechnological control tools include the transfer of resistance through grafting, the use of new sources of resistance, and the development of gene silencing strategies through the genetic transformation or CRISPR-type gene editing [13]. In addition, these novel genome editing techniques will contribute to improving our knowledge on virus–host interactions and resistance mechanisms. All approaches to engineer CRISPR virus resistance through the direct targeting of viral DNA or RNA require the permanent maintenance of CRISPR reagents in plants, which has been achieved thus far through the expression of at least one of them (Cas endonuclease) from a DNA transgene [14].

Finally, virus-induced gene silencing (VIGS), 30 years since its first application, is still a powerful tool for crop improvement. This technology is basic for the analysis of gene functions, through the downregulation of endogenous genes and the utilization of the posttranscriptional gene silencing (PTGS) machinery of plants, preventing systemic viral infections. Lee et al. [15] summarize the future of VIGS for non-model plants through the improvement of the crucial steps like proper viral vector construction and inoculation methods, as well as the development of non-transgenic crop breeding to obtain heritable epigenetic modifications; therefore, VIGS-based technologies will expand beyond gene silencing to develop virus-induced genome editing (VIGE). In this new approach, viral vectors are used to transiently deliver CRISPR components to plant cells, allowing them to be obtained in a single-generation transgene-free event. Although it is still only early days and it will be necessary to deal with difficulties that arise, it is undoubtedly a really promising and revolutionary tool for agriculture. In this sense, a recent study carried out at Berkeley University [16] has reported the successful implementation of heritable virus-induced genome editing (VIGE) in tomato. In this work, the authors developed three transgenic lines of tomato that expressed Streptococcus pyogenes Cas9 (SpCas9) under the control of Cauliflower mosaic virus 35S (35S), *S. lycopersicum* ribosomal protein S5A (SlRPS5A), or *S. lycopersicum* YAO promoters (*SlYAO*). They found that the SlYAO promoter played a critical role in driving SpCas9 expression, being fundamental to optimizing several environmental factors, such as light intensity, to improve the heritable editing frequencies.

Undoubtedly, the lower costs of mass sequencing, together with the increase in data processing capacity linked to the continuous development of CRISPR and artificial intelligence, is opening new frontiers. The discovery of new organisms (including viruses) will necessitate the reinterpretation of many diseases that have previously been associated with a virus. Therefore, the presence of other viruses, viroids, etc., is likely to lead us to rewrite virology, and it is likely that viruses are the missing link in relation to evolution, as everything seems to indicate that they were already there. Advances in new breeding targets, RNA interference (RNAi) and methylation approaches, and the application of natural antiviral compounds also present promising new strategies for effective plant virus control.
